# Developing an intrauterine device self-removal guide: a mixed methods qualitative and small pilot study

**DOI:** 10.1186/s40834-022-00177-w

**Published:** 2022-07-01

**Authors:** Francesca Collins, Kelly Gilmore, Kelsey A. Petrie, Lyndsey S. Benson

**Affiliations:** 1 University of Washington School of Public Health, 1959 NE Pacific Street Seattle, Seattle, WA 98195 USA; 2 Department of Obstetrics and Gynecology, University of Washington School of Medicine, Seattle, WA 98195 USA

**Keywords:** IUD self-removal guide

## Abstract

**Background:**

The intrauterine device (IUD) is a highly effective form of long-acting reversible contraception (LARC) with few contraindications. Users, however, often encounter barriers to desired removal. IUD self-removal may mitigate these obstacles. We sought to develop a guide for IUD self-removal with the aim of increasing user control over the method.

**Methods:**

This was a two-phase mixed-methods qualitative and small pilot study with the aim of developing an IUD self-removal guide. We conducted an online content analysis of advice for IUD self-removal as well as interviews with expert key informants to develop an IUD self-removal guide. We next recruited IUD-users who had previously attempted self-removal to participate in focus group discussion and individual interviews to further refine the guide. In the second phase of the study, we piloted the guide among eight IUD-users seeking removal interested in attempting self-removal.

**Results:**

Expert key informants agreed that IUD self-removal was safe and low risk. The primary components of successful IUD self-removal elicited were ability to feel and grasp the strings, a crouched down position, and multiple attempts. A preference for presenting IUD self-removal as safe was emphasized. In the second phase, participants in the clinical pilot suggested more information for non-palpable strings, but liked the style and information provided. One participant successfully removed their IUD.

**Conclusions:**

IUD-users reported satisfaction with our guide. In our small pilot, the majority were unable to remove their own IUD. A larger study is needed to assess acceptability, feasibility, and efficacy in increasing successful self-removal.

## Background

IUDs are highly effective forms of long-acting reversible contraception (LARC) with few contraindications. Use of IUDs has increased in the United States across demographic groups in recent years [1, 2]. IUD-users cite lack of personal control as a disadvantage as removal is often provider controlled [3]. Individuals seeking IUD removal encounter barriers including lack of provider availability, provider refusal or pressure to continue the method, cost, and lack of insurance coverage [4–7]. IUD self-removal mitigates barriers to removal and may increase reproductive autonomy.

The option of IUD self-removal has been considered as a means of increasing interest in the method with mixed results. In a survey, 25% of those seeking an abortion reported they would be more interested in an IUD with option for self-removal [8]. However, another study found that there was no difference in IUD uptake, satisfaction, or discontinuation when information of self-removal was presented attributed in part to high knowledge of IUD self-removal among the study population at baseline [9]. There is indeed a great deal of information about IUD self-removal available to the public online. A recent separate content analysis found that 58 online videos discussing LARC self-removal had nearly 4 million views [10]. One multi-site study evaluated interest and experiences with IUD self-removal. While only 19% of participants successfully removed their own IUD, the majority would recommend self-removal to a friend (58%) and attempt self-removal in the future (54%) [11]. The study utilized a one-page guide for self-removal instructing participants to wash their hands with soap, find a position to best reach the strings, and to pull the strings gently and firmly noting that the IUD should come out with a gentle tug [11].

We aimed to develop a guide rooted in the advice and experience of IUD users who had previously attempted self-removal and key expert informant opinion. While there is certainly information available online about self-removal, we sought to create a single consolidated and comprehensive resource in an easily accessible and user-friendly format using gender neutral terminology and graphics. We completed the work to develop this guide prior to the COVID-19 pandemic and it has since become increasingly relevant. Notably, in the early stages of the pandemic, the American College of Obstetricians and Gynecologists discouraged LARC removal instead advising, "Existing IUD and implant users who seek removal and replacement of their contraceptives should be counseled about extended use of these device" [12]. These new challenges to accessing comprehensive reproductive healthcare underscore the importance of a comprehensive tool to assist in IUD self-removal as a means of increasing reproductive autonomy.

## Methods

**Fig. 1:**
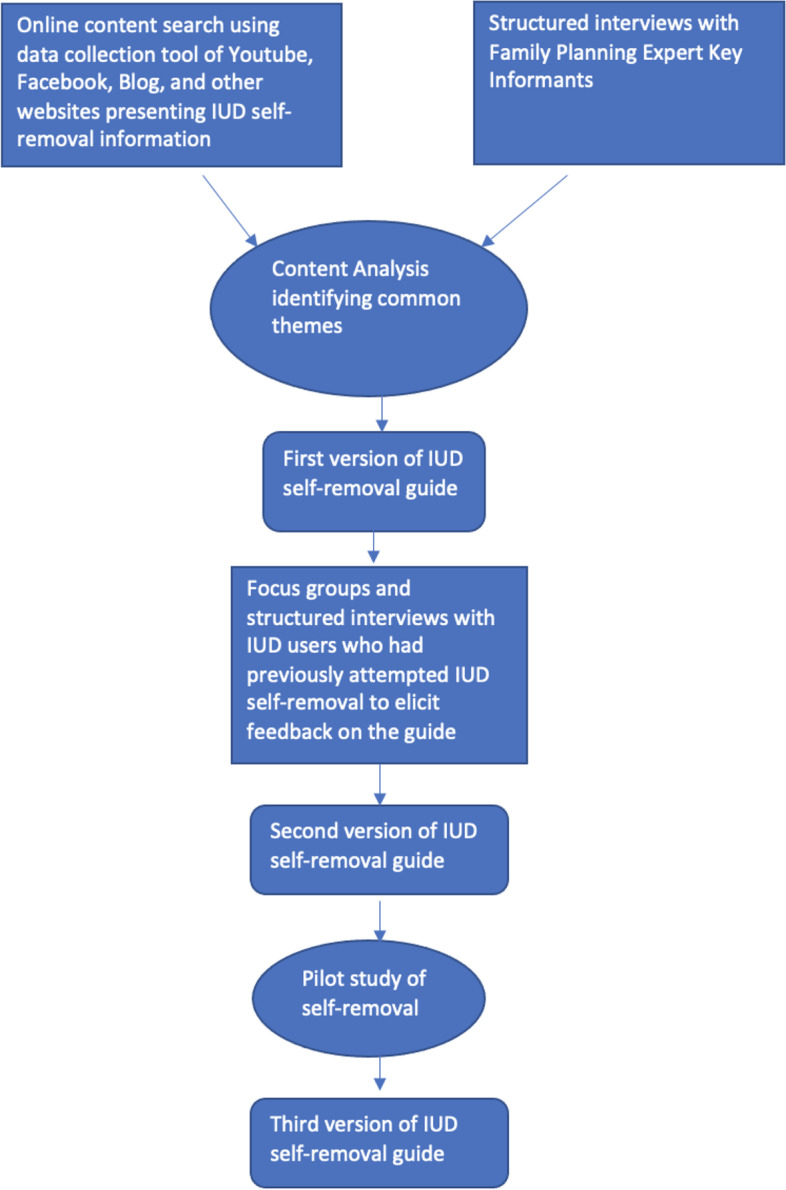
Guide development flow

This was a two-phase mixed-methods qualitative and small pilot study that took place from July 2016-December 2017. All phases were approved by the University of Washington Human Subjects Division. The first phase of this study had two parts. First, using an online content search and expert key informant interviews an initial version of the IUD self-removal guide was developed. Second, IUD users who had previously attempted self-removal were recruited to participate in focus groups or individual interviews about their experience attempting self-removal and thoughts on our IUD self-removal guide. Based on their feedback, the guide was adapted and then piloted in a small convenience sample in phase two. The phases of the study are summarized in Fig. 1.

### Phase I: qualitative development of an IUD self-removal guide

The first phase included first both an online content search and expert key informant interviews to develop an initial version of the self-removal guide followed by interviews with IUD users who had previously attempted self-removal for further refinement. We developed a tool to collect data from women presenting information and advice on IUD self-removal online from YouTube, Facebook, personal blogs, and websites. The tool collected information on the reason for self-removal, a description of the process they tried to self-remove, an indication of whether they had ever felt their strings before, their advice to others wishing to attempt self-removal, whether they were successful, and an analysis of the comments posted and whether they indicated viewers/readers found the advice helpful or unhelpful.

For our expert key informants, we recruited family planning providers and experts in the Seattle area using personal connections and snowball sampling. Key informants were recruited from community-based clinics, academic medical centers, private OB/GYN practices, the public health department, Planned Parenthood, midwifery practices, and reproductive and sexual health advocacy and research non-profits. We used a semi-structured interview guide for key informants that covered their professional opinion on the safety and community need for IUD self-removal, their experience with patients who have attempted IUD self-removal, their advice for instructing women to remove their own IUDs, their concerns about risks and safety, and their advice on how to best present information on self-removal.

We analyzed data from the online search and interviews using a content analysis approach identifying common themes in Dedoose qualitative software to develop the first version of the self-removal guide.

### Phase I: refinement of the developed IUD self-removal guide

After using the qualitative data from the online content search and key expert informant interviews to develop a first version of the IUD self-removal guide, we presented this version of the self-removal guide to IUD-users who had attempted self-removal for further refinement in focus groups and semi-structured interviews. We recruited IUD users who had previously tried to remove an IUD via online, print and social media advertisements to participate in a focus group or interview about their experience. Women had to be English-speaking, 18 years of age or older and had ever attempted to remove their own IUD; they did not need to be successful in their attempt. In both settings, we asked the same semi-structured questions covering reasons for attempting IUD self-removal, experience with self-removal, concerns prior to attempting self-removal, and feedback on the guide. Participants were given a $35 gift card.

We analyzed focus group and interview transcripts in Dedoose qualitative software. Two members of the research team developed an a priori codebook from focus group and interview transcripts that was applied to all transcripts to elucidate common themes. Our initial goal was to reach up to 30 research participants, however, we reached thematic saturation after hearing from eight participants about their IUD self-removal attempts. From this analysis we designed version two of the self-removal guide incorporating their comments on their experience with self-removal and direct feedback on the guide.

### Phase II: piloting the self-removal guide

We approached patients seeking IUD removal at the University of Washington Family Planning Clinic. After providing informed consent, IUD-users completed a pre-study questionnaire. They were then given 10–15 min alone in an exam room to attempt self-removal using the online guide. Patients could indicate they were done attempting self-removal at any time. If the patient was unsuccessful, the physician entered and removed the IUD. Participants then completed the post-study questionnaire about whether they were successful, their level of pain, and their feedback on the guide. Providers recorded if participants experienced any adverse events. Participants were given $35 for their time.

## Results

We collected data from six websites where IUD-users shared their own successful IUD self-removal experiences. We recruited 12 expert key informants (three public health professionals, a nurse practitioner, four midwives, and four physician expert key informants). Three primary components of successful IUD self-removal emerged from this analysis: 1) ability to feel and grasp the strings 2) a crouched down position that included “bearing down” 3) multiple attempts using different body positions.

Expert key informants agreed that IUD self-removal was safe and low risk. They recommended those interested in IUD self-removal wash their hands, feel for their strings, and pull down and out firmly. Three medical professionals suggested wearing gloves to better be able to grip the strings. All twelve suggested including information about need for a different method of birth control if fertility was not desired.

We then held one focus group with five participants and three in-depth interviews with individuals unable to attend the focus group, participant demographics are summarized in Table 1. They indicated a preference for an online guide over a pamphlet. Participants indicated a preference for presenting self-removal without overcomplication or repeat warnings to seek medical care. The most commonly stated reasons for IUD self-removal were desired fertility, cost, and long appointment wait times.

**Table 1:** Demographics of focus group and interview participants with prior self-removal attempt

Variable	% (N)
Age	
18–29	13 (1)
30–39	63 (5)
40–49	13 (1)
50–59	13 (1)
Race/Ethnicity	
White	75 (6)
Black	-
Hispanic/Latinx	13 (1)
Asian/Pacific Islander	13 (1)
Native American	-
Highest level of Education	
High School	13 (1)
Some College	13 (1)
Associate’s Degree	13 (1)
Bachelor’s Degree	38 (3)
Master’s Degree	25 (2)
Advanced Graduate Degree	-
Annual Household Income	
Under $25,000	-
$25,000—$39,000	-
$40,000—$50,000	38 (3)
$50,000—$75,000	25 (2)
$75,000—$100,000	13 (1)
Over $100,000	13 (1)

**Fig. 2:**
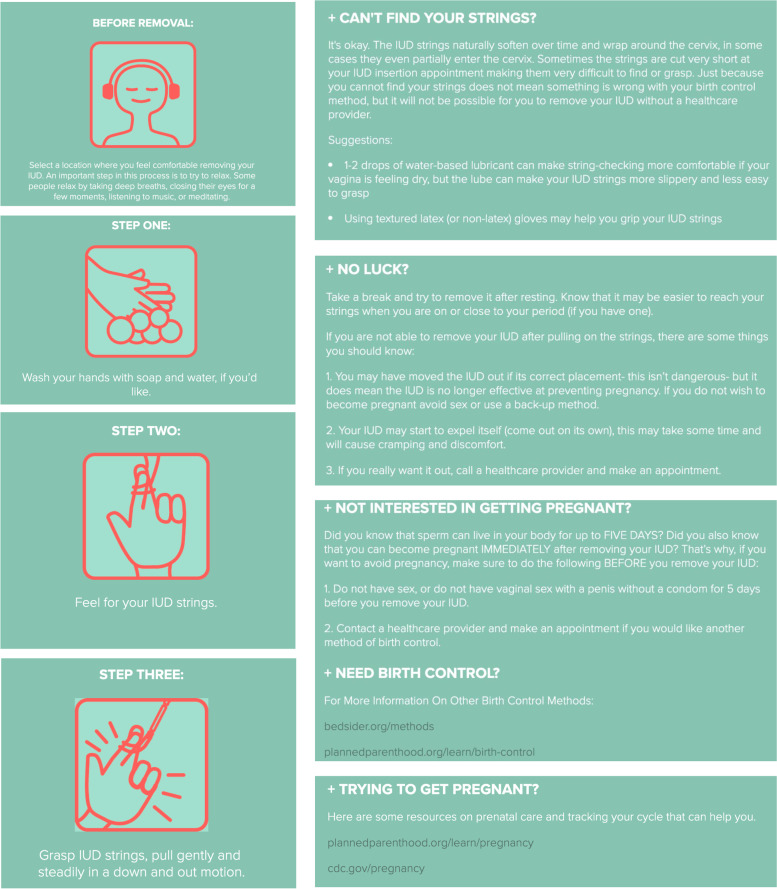
IUD self-removal guide website representative content, IUD-self removal steps with example of trouble-shooting

Version two of the IUD self-removal guide was created as a website in collaboration with a graphic design student with a short infographic video and step-by-step process. Representative images are included as Fig. 2. Eight IUD-users desiring removal participated in the clinical pilot using this version, their demographics are summarize in Table 2.

**Table 2:** Demographics of pilot participants

Variable	% (N)
Race/Ethncitiy	
White	86 (6)
Black	-
Hispanic/Latinx	-
Asian/Pacific Islander	-
Native American	14 (1)
Highest Level of Education	
High School	-
Some College	14 (1)
Associate’s Degree	-
Bacehelor’s Degree	43 (3)
Advanced Graduate Degree	43 (3)
Annual Household Income	
Under 25,000	14 (1)
$25,000 – 39,000	-
$40,000 – 50,000	-
$50, 000 – 75,000	14 (1)
$75,000 – 100,000	43 (3)
Over $100,000	29 (2)

All pilot participants indicated that they liked the guide, found it helpful and easy to understand, and liked the multimedia elements including the video. When asked what they liked best about the guide, the majority of participants cited the simplicity, the icons, and the video. They suggested more tips on finding and gripping the strings. One participant was successful in removing their IUD. The remaining participants had their IUDs removed by providers. String lengths were records and the average length was 5.8 cm. All participants reported that they felt “somewhat comfortable” or “comfortable” attempting IUD self-removal. There were no adverse events.

## Discussion

We developed an online IUD self-removal guide using input from IUD-users who had successfully removed their own IUDs and expert key informants. The primary components of successful IUD self-removal elicited were ability to feel and grasp the strings, a crouched down position, and multiple attempts. A preference for presenting IUD self-removal as safe was emphasized. We then piloted our guide among a small convenience sample in our clinic. Participants in our small clinical pilot liked the overall design, feel, and content of the online IUD self-removal guide. They suggested including more advice on finding IUD strings, feedback we have used to further refine the guide.

Currently, the online IUD self-removal guide is a website containing an introduction to IUD self-removal; disclaimers for those interested in attempting; a video animation with a step-by-step guide; a page dedicated to frequently asked questions including trouble-shooting for non-palpable strings; information on birth control options; and resources for those desiring pregnancy after removal. It consolidates the advice from content analysis of IUD-users who have previously attempted self-removal and key expert informants to present IUD self-removal as safe and feasible. It presents this information with gender neutrality.

Participants in our small clinical pilot liked the overall design, feel and content of the online IUD self-removal guide but the majority were unable to self-remove their IUD. Our rates of successful self-removal (12.5%) are lower than previously found (19%) [10], however we were limited by a very small convenience sample size. Further, within our small convenience sample, the average string length was lower than in the work by Foster et al. (5.8 cm versus 6.7 cm), who also found a 50% increase in success rate of IUD self-removal with each 0.5 cm increase in string length.

The strengths of our study include the mixed methods to incorporate available online content as well as iterative feedback of key expert informants and IUD-users. Weaknesses of our study include the small sample size and convenience sample of those scheduled for IUD removal in clinic. Additionally, our expert key informants and pilot participants were primarily white, well educated, and in their 30’s.

## Conclusions

A larger study of the online IUD self-removal guide is needed to evaluate feasibility, acceptability, and effectiveness among a diverse group of IUD-users seeking self-removal information including those in a non-clinical setting. Ultimately, a simple resource aiding IUD-users in self-removal may be used as a tool to increase reproductive autonomy and to normalize patient-provider conversations around self-removal.

## Data Availability

Upon request.

## Abbreviations

LARC: Long acting reversible contraception
IUD: Intrauterine Device

## References

[1] Kavanaugh ML, Pliskin E. Use of contraception among reproductive-aged IUD-users in the United States, 2014 and 2016. F&S Reports. 2020;1(2):83–93. https://www.fertstertreports.org/article/S2666-3341(20)30038-6/fulltext. 10.1016/j.xfre.2020.06.006 PMC8244260 34223223

[2] Finer LB, Jerman J, Kavanaugh ML (2012). Changes in use of long-acting contraceptive methods in the United States, 2007–2009. Fertil Steril.

[3] Asker C, Stokes-Lampard H, Beavan J, Wilson S (2006). What is it about intrauterine devices that IUD-users find unacceptable? Factors that make IUD-users non-users: a qualitative study. J Fam Plann Reprod Health Care.

[4] Amico JR, Stimmel S, Hudson S, Gold M (2020). "$231 … to pull a string!!!" American IUD-users' reasons for IUD self-removal: an analysis of internet forums. Contraception.

[5] Amico JR, Bennett AH, Karasz A, Gold M (2016). "She just told me to leave it": IUD-users's experiences discussing early elective IUD removal. Contraception.

[6] Amico JR, Bennett AH, Karasz A, Gold M (2018). Taking the provider "out of the loop:" patients' and physicians' perspectives about IUD self-removal. Contraception.

[7] Armstrong E, Gandal-Powers M, Levin S, Kimber-Kelinson A, Luchowski A, Thompson K. Intrauterine devices and implants: a guide to reimbursement. https://larcprogram.ucsf.edu/. July 2015. Accessed 9/8/21.

[8] Foster DG, Karasek D, Grossman D, Darney P, Schwarz EB (2012). Interest in using intrauterine contraception when the option of self-removal is provided. Contraception.

[9] Raifman S, Barar R, Foster D (2018). Effect of knowledge of self-removability of intrauterine contraceptives on uptake, continuation, and satisfaction. Womens Health Issues.

[10] Kathleen Broussard, Andréa Becker. Self-removal of long-acting reversible contraception: A content analysis of YouTube videos. Contraception. 2021;104(6):654–658. 10.1016/j.contraception.2021.08.002. Epub 2021 Aug 13. PMID: 34400154; PMCID: PMC8592268. 10.1016/j.contraception.2021.08.002 PMC8592268 34400154

[11] Foster DG, Grossman D, Turok DK, Peipert JF, Prine L, Schreiber CA, Jackson AV, Barar RE, Schwarz EB (2014). Interest in and experience with IUD self-removal. Contraception.

[12] AM. Kaunitz. COVID-19 gynecology practice recommendations from ACOG**.** NEJM J Watch Womens Health; 2020. https://www.jwatch.org/na51312/2020/04/16/covid-19-gynecology-practice-recommendations-acog. Accessed 30 Nov 2021.

